# Saturation and self-absorption effects in the angle-dependent 2*p*3*d* resonant inelastic X-ray scattering spectra of Co^3+^

**DOI:** 10.1107/S1600577520005123

**Published:** 2020-06-09

**Authors:** Ru-Pan Wang, Hebatalla Elnaggar, Charles J. Titus, Keisuke Tomiyasu, Jaap Geessinck, Gertjan Koster, Federica Frati, Jun Okamoto, Di-Jing Huang, Frank M. F. de Groot

**Affiliations:** a Debye Institute for Nanomaterials Science, Utrecht University, Universiteitsweg 99, 3584 CG Utrecht, The Netherlands; b Department of Physics, Stanford University, Stanford, CA 94305, USA; c Department of Physics, Tohoku University, Aoba, Sendai 980-8578, Japan; d NISSAN ARC Ltd, 1 Natsushima-cho, Yokosuka, Kanagawa 237-0061, Japan; e MESA+ Institute for Nanotechnology, University of Twente, 7500 AE Enschede, The Netherlands; f Condensed Matter Physics Group, National Synchrotron Radiation Research Center, 101 Hsin-Ann Road, Hsinchu Science Park, Hsinchu 30076, Taiwan, Republic of China

**Keywords:** angle-dependent RIXS/RXES, saturation and self-absorption effects

## Abstract

Angle-dependent 2*p*3*d* resonant inelastic X-ray scattering spectra of a LaCoO_3_ single crystal and a 55 nm LaCoO_3_ film on a SrTiO_3_ substrate are presented. Theoretical calculation shows that, with ∼20 meV resolved Co 2*p*3*d* resonant inelastic X-ray scattering (RIXS), the excited states of the isotropic ^1^A_1*g*_(O_*h*_) ground state are split by 3*d* spin–orbit coupling, which can be distinguished via their angular dependence. However, strong self-absorption and saturation effects distort the spectra of the LaCoO_3_ single crystal and limit the observation of small angular dependence. In contrast, the RIXS on 55 nm LaCoO_3_ shows less self-absorption effects and preserves the angular dependence of the excited states.

## Abstract 2

It is shown that the 2*p*3*d* resonant inelastic X-ray scattering intensity is distorted by saturation and self-absorption effects, *i.e.* by incident-energy-dependent saturation and by emission-energy-dependent self-absorption.

## 1. Introduction   

Resonant inelastic X-ray scattering (RIXS) is a developing technique that is used to investigate the ground and excited states of transition metals, in particular when combined with the angular degrees of freedom (*i.e.* angle-dependent measurements). The angular dependence of RIXS has been examined by Michel van Veenendaal using crystal field calculations for *L*- and *M*-edges in the case of a single valence hole (or equally a single electron) (van Veenendaal, 2006 ▸). Based on these calculations, one is able to discriminate between different transitions of the *d*-states via their spin characteristics using certain photon polarizations or experimental geometries for RIXS (van Veenendaal, 2006 ▸; Moretti Sala *et al.*, 2011 ▸). By including the spin–orbit coupling, exchange field (2*J*_ex_) or external magnetic field, the system symmetry is further reduced (van Schooneveld *et al.*, 2012 ▸). This implies that RIXS can even have the sensitivity to investigate the fine structure of spin–orbit coupling in multi-electron systems. However, this sensitivity is commonly hampered by geometrical aspects of the experiment.

The RIXS cross-section can be explained as a combination of an absorption (*photon-in*) process and an emission (*photon-out*) process. The absorption process excites the electrons to higher energy levels and the emission process emits photons when the electrons decay to the lower energy levels. Two geometrical effects have to be taken into account here: (i) the probing depth is dependent on the X-ray absorption spectroscopy (XAS) cross-section (saturation) and (ii) the emitted photons can be re-absorbed (self-absorption). Consequently, the emission intensity might be distorted according to the photon energy and the experimental geometry, which is well known in fluorescence yield XAS (FY-XAS) (Jaklevic *et al.*, 1977 ▸; Zschech *et al.*, 1992 ▸; Tröger *et al.*, 1992 ▸; Eisebitt *et al.*, 1993 ▸; Chakarian *et al.*, 1998 ▸; Nakajima *et al.*, 1999 ▸; Achkar *et al.*, 2011 ▸). As such, it also distorts the RIXS spectra (Chabot-Couture *et al.*, 2010 ▸; Dallera *et al.*, 1997 ▸).

In this article, we raise awareness that the 2*p*3*d* RIXS intensity is distorted not only by the incident-energy-dependent saturation but also by emission-energy-dependent self-absorption. A model is proposed to describe the angle-dependent coefficients of the RIXS intensity for the saturation and self-absorption effects, which has been used to correct the 2*p*3*d* RIXS intensity of magnons in cuprate (Minola *et al.*, 2015 ▸), where we applied the self-absorption-added coefficients to generate a theoretical prediction. The angle-dependent 2*p*3*d* RIXS of a LaCoO_3_ single crystal and a 55 nm LaCoO_3_ film on an SrTiO_3_ substrate (LaCoO_3_/SrTiO_3_ film) were compared to illustrate these effects. Furthermore, we show that the fine structure of the spin–orbit coupling and exchange interaction within the 3*d* ^6^ manifold of a Co^3+^ ion can potentially be revealed using RIXS angle-dependent measurements.

## 2. Theory   

### 2.1. Kramers–Heisenberg formula   

The RIXS cross-section is determined by the Kramers–Heisenberg equation (Kramers & Heisenberg, 1925 ▸),
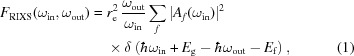
where *A*_*f*_(ω_in_) is the scattering amplitude which is defined as
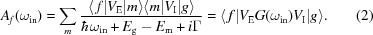
Here ℏω_in_ and ℏω_out_ are the energies of the incident and the emitted photons; *E*_g_, *E*_f_ and *E*_m_ are the eigenvalues of the ground state |*g*〉, the final state |*f*〉 and the intermediate state |*m*〉, respectively; Γ gives the lifetime broadening of the intermediate state. *V*_I_(*V*_E_) is the transition operator induced by the electro-magnetic field of the incident (emitted) photon and can be expanded as 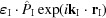 (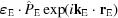). **∊**_I_ (**∊**_E_) and 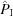 (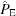) describe the electric field polarization vector and the momentum operator of the incident(emitted) photon. **k**_I_ (**k**_E_) and **r**_I_ (**r**_E_) give the wavevector and position of the electro-magnetic wave. For 2*p*3*d* RIXS, the 2*p*3*d* (3*d*2*p*) transition operator is approximated as an electric dipole operator 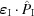 (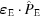) for the absorbed (emitted) channel, *i.e.* 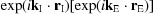 ≃ 1. The scattering amplitude can also be described by Green’s function [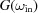 = 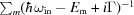], where the ground-state energy (*E*_g_) is taken as zero. Note that we can ignore the scattering coefficient 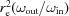 because 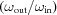 ≃ 1 (ω_in_ ≃ ω_out_) and the classical electron radius *r*_e_ is constant.

**Figure 1:**
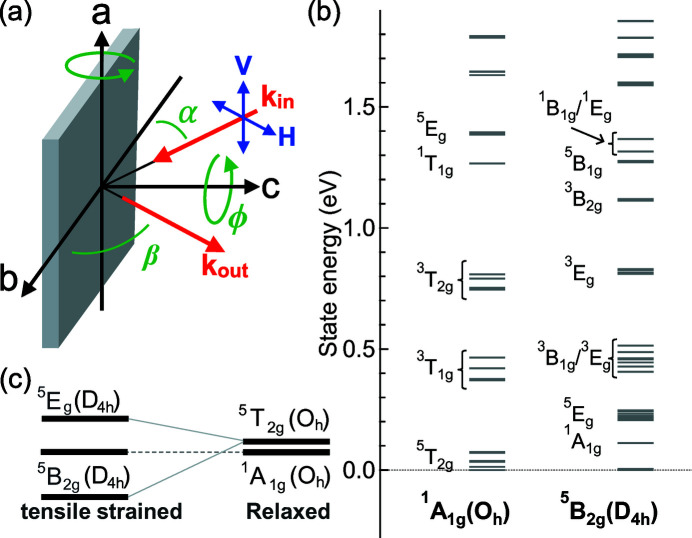
(*a*) Illustration of the geometry and the angles. (*b*) Energy diagram for the ^1^A_1*g*_(O_*h*_) ground state and the ^5^B_2*g*_(D_4*h*_) ground state. (*c*) The ^5^B_2*g*_(D_4*h*_) state is the split subgroup of the ^5^T_2*g*_(O_*h*_) state in the tetragonal distortion (in-plane tensile strained) (Wang *et al.*, 2019 ▸).

For a better insight into the angular dependence of RIXS, many authors have already reformulated the scattering amplitude by using the spherical tensor expansion (Luo *et al.*, 1993 ▸; van Veenendaal, 2006 ▸; Juhin *et al.*, 2014 ▸; Moretti Sala *et al.*, 2011 ▸). In the case of the 2*p*3*d* RIXS within the fast-collision approximation, the interference of the intermediate states can be ignored and Green’s function can be simplified by *G* ^±^(ω_in_) 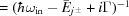, where 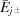 is the mean energy for the spin–orbit coupled 2*p*_3/2_ and 2*p*_1/2_ edges (Luo *et al.*, 1993 ▸; van Veenendaal, 2006 ▸). Then the 2*p*3*d* RIXS cross-section formula can be expanded as
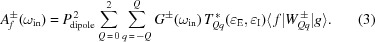
Van Veenendaal pointed out that the cross-section is now described by the effective *dd* transition operators *W*_*Qq*_ from a 3*d* ^*n*^ ground state to another 3*d* ^*n*^ final state with a polarization weighting factor *T*_*Qq*_(∊_E_,∊_I_) (van Veenendaal, 2006 ▸). *W*_*Qq*_ is a one-particle operator which is constructed by the orbital- and spin-dependent tensors. The system geometry [Fig. 1(*a*) ▸] is reflected by the polarization tensors *T*_*Qq*_(∊_E_,∊_I_). *P*_dipole_ is the reduced matrix element between the 2*p* (core) and 3*d* (valence) levels. With this simplification, the beam polarization and the electronic configuration can be decoupled from each other and they therefore can be linked directly to the experimental geometry.

In this work, the ground and excited states’ energies and the 2*p*3*d* RIXS spectra were computed using the program *Quanty*, where both the tetragonal distortion and ligand-to-metal charge transfer could be considered (Haverkort *et al.*, 2012 ▸; de Groot, 2005 ▸). The polarization operators were expanded on the coordinates of the crystal field operator, where the axes are given by **a**, **b** and **c** as indicated in Fig. 1(*a*) ▸. Because no polarization analyzer was used, unpolarized emitted photons have been assumed and were composed by the summation of two orthogonal linear polarized beams (Juhin *et al.*, 2014 ▸). The energy diagrams of the final (excited) states |*f*〉 were obtained from the cluster calculation using the program *Quanty* (Haverkort, 2010 ▸). The exchange interaction is estimated to be 6 meV (*T*_c_ ≃ 70 K) which is neglected in the current simulation and discussion since it shows only tiny effects on the spectra. The electronic configurations of Co^3+^ ions in the LaCoO_3_ crystal and the LaCoO_3_/SrTiO_3_ film were suggested to be the pure ^1^A_1*g*_(O_*h*_) configuration and a mixture of the ^1^A_1*g*_(O_*h*_) and the ^5^B_2*g*_(D_4*h*_) configurations, respectively (Tomiyasu *et al.*, 2017 ▸; Wang *et al.*, 2019 ▸). The model parameters of the ^1^A_1*g*_(O_*h*_) ground state configuration are: 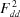 = 9.371 eV, 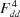 = 5.859 eV, ζ_*d*_ = 0.055 eV, 10*Dq* = 0.595 eV, *U* = 6.5 eV, *Q* = 7.5 eV, Δ = 1.5 eV, 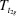 = 1.8 eV and 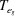 = 3.118 eV. For the ^5^B_2*g*_(D_4*h*_) ground state configuration, *Ds* = −0.12 eV and *Dt* = −0.018 eV are applied in addition to the ^1^A_1*g*_(O_*h*_) case. The calculated excited states are indicated by the term symbol notations in Fig. 1(*b*) ▸. We note that the ^5^B_2*g*_(D_4*h*_) state is the subgroup of the ^5^T_2*g*_(O_*h*_) state in the tetragonal distortion [Fig. 1(*c*) ▸].

### 2.2. Saturation and self-absorption effects   

**Figure 2:**
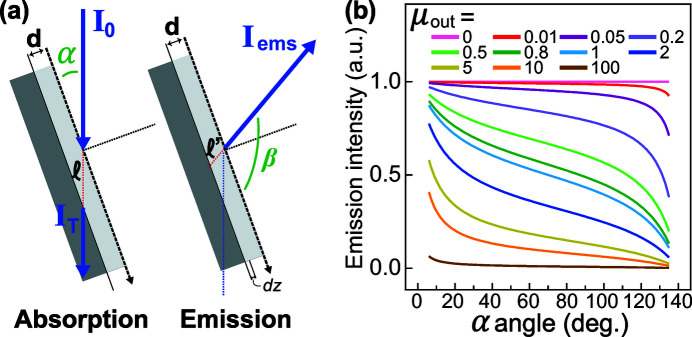
(*a*) Illustration of the geometry for the absorption and emission process. (*b*) Self-absorption-added coefficients {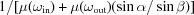} as a function of the sample rotation angle α, where μ(ω_in_) = 1 and μ(ω_out_) is scaled from zero to infinity to simulate the trends. The scattering angle (α + β) is set to 140°.

To simulate the saturation and the self-absorption effects, the transmitted photons (*I*_T_) within a small distance dℓ can be described by the differential Lambert–Beer relation with the absorption factor of the incident beam μ(ω_in_),
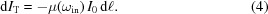
The integration of equation (4) from zero to the path length ℓ becomes:
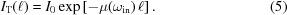
Here the constant *I*_0_ gives the finite incoming photon flux. We note that this relation also depends on the surface profile of the sample. For a flat sample, the absorption probing depth *d* depends on the angle α between the incident photon and the sample surface [Fig. 2(*a*) ▸], which gives the relation 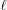 = 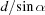. Thus we can rewrite equation (5) as a function of *d*,
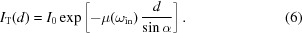
For the emission process, the total emitted flux is the integration of the beam of emitted photons from the surface to the probing depth *d*. This implies the path length 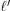 = 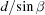, where β is the angle between the scattered beam and sample surface [Fig. 2(*a*) ▸]. Then the total emitted flux becomes
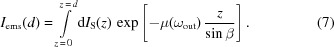
Here, d*I*_S_(*z*) stands for the scattering possibility per absorbed photon within a finite distance d*z* at a certain depth *z*, from which we can expand d*I*_S_(*z*) into equation (4) with a scattering possibility *S*(ω_in_,ω_out_) as
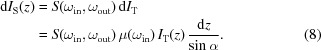
The scattering possibility *S*(ω_in_,ω_out_) is determined by the state transition possibility. To a good approximation, the multiplication *S*(ω_in_,ω_out_) μ(ω_in_) represents the scattering intensity of RIXS. We note that our simulation calculated the scattering intensity of RIXS including the interference effects. In addition, the Auger decay channels are also state dependent. This will induce additional scaling factors on the emission intensity. In the case of the 2*p*3*d* channels, the energy dependence in Auger decay is less than 10% (de Groot *et al.*, 1994 ▸) and we approximate them as constant. By integrating equation (7), the emission intensity including both the saturation and the self-absorption effects is written as (as a function of probing depth *d*)
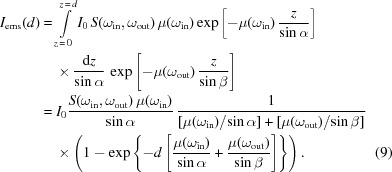
This equation has already been discussed for the cases of FY-XAS spectra (Jaklevic *et al.*, 1977 ▸; Zschech *et al.*, 1992 ▸; Tröger *et al.*, 1992 ▸; Eisebitt *et al.*, 1993 ▸; Chakarian *et al.*, 1998 ▸; Nakajima *et al.*, 1999 ▸; Achkar *et al.*, 2011 ▸; Chabot-Couture *et al.*, 2010 ▸) by assuming that μ(ω_out_) is approximately constant. The spectral saturation is described by the term 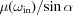 and is sensitive to the incidence angle α; the self-absorption effect is determined by the emission angle β. For RIXS, however, μ(ω_out_) cannot be treated as a constant because the emitted photon energy is analyzed.

#### 2.2.1. Thin samples   

If the sample thickness (*D*) is much smaller than the attenuation length (η), *i.e.* η ≫ *D* ≃ *d* → 0, we can use the approximation of the exponential function, exp(−*x*) ≃ 1 − *x*. Then we obtain
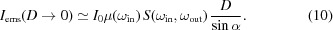
Equation (10) shows that the emission intensity is proportional to the incoming photon flux *I*_0_, the absorption factor of the incident beam μ(ω_in_), the scattering factor *S*(ω_in_,ω_out_), the sample thickness *D* and the angle α. Thus, the total emission intensity only depends on the rotation angle α, which changes the overall intensity as a function of angle but shows no energy-dependent spectral distortion caused by the self-absorption effect. This self-absorption-free approximation is also applied to dilute specimens. μ(ω_in_) and μ(ω_out_) are the total absorption factors at certain energies ω_in_ and ω_out_, which can also be described by the summation over all individual elements ‘*X*’, *i.e.* 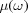 = 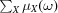 and 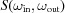 = 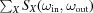. The FY-XAS or RIXS intensity is proportional to the multiplication *S*(ω_in_,ω_out_) μ(ω_in_). By ignoring the inter-atom interaction, this multiplication of the two summation factors μ(ω)*S*(ω_in_,ω_out_) is replaced by the summation of the multiplications 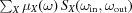. Then equation (10) is expressed as
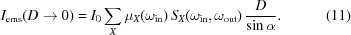
In the case of the Co *L*_3_ edge, the absorption factor and scattering possibility of the other elements are constant so that equation (11) becomes
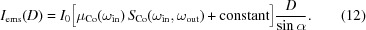
The state-dependent scattering possibility *S*_Co_(ω_in_,ω_out_) implies that the partial fluorescence yield X-ray absorption is not identical to the normal absorption. Liu *et al.* show good agreement of such state-dependent behavior between experimental and calculated results in dilute specimens (Liu *et al.*, 2018 ▸). If *S*_Co_(ω_in_,ω_out_) is approximated as a constant, the scattered spectrum is equivalent to the normal absorption μ_Co_(ω_in_).

#### 2.2.2. Thick samples   

If the sample is thick (large *D*), *d* becomes infinity; the incident photons are fully absorbed. Thus the exponential term in equation (9) becomes zero. Then we obtain the scattering intensity as a function of the photon energy and sample geometry,
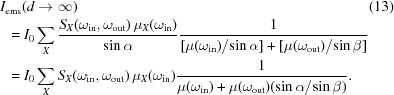
This relation has been applied to a method of inverse partial fluorescence yield to obtain bulk-sensitive absorption spectra (Achkar *et al.*, 2011 ▸), where the key aspect of inverse partial fluorescence yield is that one applies the partial fluorescence yield away from the target edges, so that the multiplication of μ_*X*_(ω_in_) and *S*_*X*_(ω_in_,ω_out_) is approximately constant. For example, in CoO, the element cobalt and the element oxygen are considered. Then the emission intensity becomes
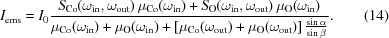
When accumulating the oxygen *K* edge fluorescence signal, the emission intensity of cobalt is negligible [μ_Co_(ω_in_)*S*_Co_(ω_in_,ω_out_) ≃ 0]. The emission spectrum becomes the summation of the emitted photon energy (ω_out_) at the oxygen *K* edge, which leads to μ_O_(ω_in_)*S*_O_(ω_in_,ω_out_) that is approximated as constant with respect to the incident energy of the cobalt *L*_2,3_ edge. It also implies that the absorption factors of the emitted photons [μ_Co_(ω_out_) and μ_O_(ω_out_)] are approximately constant. Then the emission intensity is simplified as
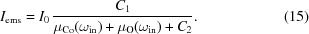
The inverse intensity becomes 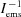 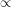 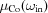 + 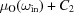, where *C*_1_, *C*_2_ and μ_O_(ω_in_) are constant with respect to the incident energy of the cobalt *L*_2,3_ edge. Thus the emission intensity is mainly dominated by the absorption factor μ_Co_(ω_in_) which provides a bulk-sensitive absorption spectrum free from self-absorption and saturation effects.

For RIXS, equation (13) shows that the scattering intensity of RIXS should be multiplied by the self-absorption-added coefficients 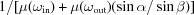 when saturation and self-absorption effects are included (Chabot-Couture *et al.*, 2010 ▸). Fig. 2(*b*) ▸ shows this saturation relation as a function of α. Here, μ(ω_in_) is fixed at one and μ(ω_out_) is scaled from zero to a hundred (infinity) to simulate the trends. The scattering angle is 140°. Depending on the competition between μ(ω_in_) and μ(ω_out_), three cases can occur:

(i) μ(ω_out_) ≫ μ(ω_in_) (*I*_ems_ ≃ 0). In this condition, the absorption factor of the emitted photons is much bigger than the incident beam absorption. The scattered photons can more easily be reabsorbed in the emission process, which implies that the emission intensity tends to vanish [the line μ(ω_out_) ≃ 100 in Fig. 2(*b*) ▸].

(ii) 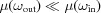 (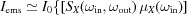/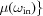). When the absorption factor of the emission beam is much smaller than the incident beam, the angle-dependent term is negligible together with the small μ(ω_out_) value. In other words, the spectrum distortion is angle-independent [the line μ(ω_out_) ≃ 0 in Fig. 2(*b*) ▸].

(iii) μ(ω_out_) ≃ μ(ω_in_). In this case, none of the factors can be omitted, as shown by the lines μ(ω_out_) ≃ 1. The self-absorption effect is dependent on the geometry and the photon energy of both the incident and emitted beams. From the equation, we specify the two extreme conditions. The first case is that the incident beam is propagating along the sample surface plane (grazing incidence), where α ≃ 0. Here, the emission intensity 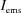 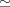 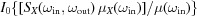 provides a maximum value. But the absorption cross-section is saturated due to the competition between the absorption factor on certain element μ_*X*_(ω_in_) and the total absorption factor μ(ω_in_). The second case describes the propagation of the emitted beam being along the sample surface plane (grazing exit). It implies that the scattered photons are totally reabsorbed and no scattering intensity should be expected (*I*_ems_ ≃ 0).

**Figure 3:**
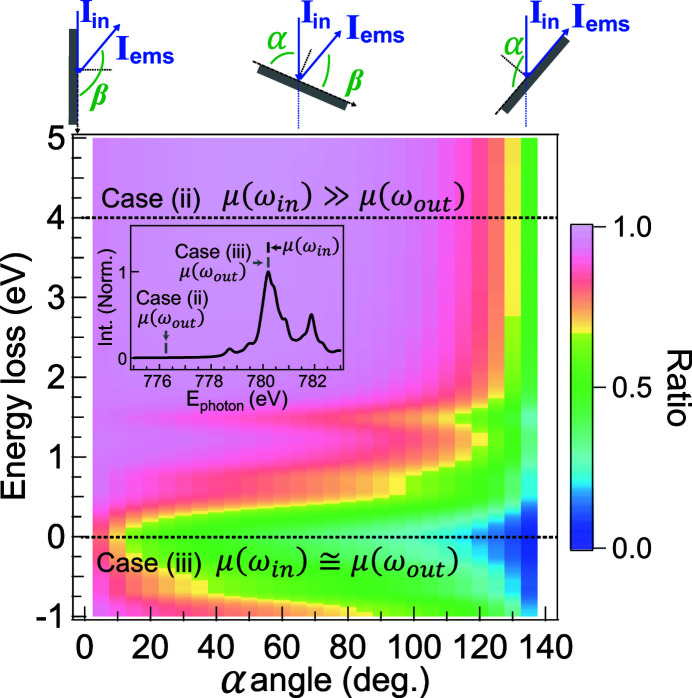
The self-absorption-added coefficients {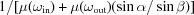} as a function of rotation angle and emitted photon energy loss for a low-spin Co^3+^ ^1^A_1*g*_(O_*h*_) ground state, where α + β = 140°. The absorption factor μ is plotted in the inset. It indicates also the conditions of case (ii) μ(ω_in_) ≫ μ(ω_out_) and case (iii) μ(ω_in_) ≃ μ(ω_out_). We note that the absorption factor has been normalized to the maximum and the self-absorption-added coefficients have been normalized to the maximum of the region of interest.

We present in Fig. 3 ▸ the influence of the self-absorption and saturation effects on the low-spin Co^3+^ ^1^A_1*g*_(O_*h*_) ground state as an example for bulk LaCoO_3_. The theoretical μ_Co_(ω_out_) and μ_Co_(ω_in_) can be obtained by calculating the XAS spectra. μ_Co_(ω_out_) is approximately equal to the total μ_Co_(ω). For a fixed incident photon energy, the incident absorption factor μ_Co_(ω_in_) is always the same. Note that the total μ(ω) includes not only the contribution of the target element but also the contributions from other elements. We assume that the other absorption channels result in a constant background μ_B_ of about 5% of the total μ(ω) (the value is estimated in Appendix *A* [App appa]). Fig. 3 ▸ shows the self-absorption-added coefficients {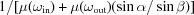} as a function of rotation angle and energy loss (energy transfer). The emitted signal can be classified into two parts: the *dd* excitations and the fluorescence. The emitted photons’ energies of *dd* excitations are close to the *L*_3_ edge absorption, which implies that 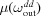 ≃ 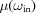, so case (iii) is applied. In contrast, the emission energy of the fluorescence feature is ∼2.5 eV lower than the elastic peak for the main line of *L*_3_ RIXS, so it experiences considerably less absorption. According to the calculation, the absorption factor 2.5 eV before the edge is considerably smaller. We can assume that 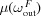 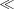 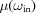 which leads to case (ii) and shows no angular dependence. This shows that the overall intensity of the angle-dependent spectra can be normalized to the fluorescence feature.

## 3. Methodology   

The single crystal of LaCoO_3_ was prepared from the polycrystalline sample which was a stoichiometric mixture of high-purity powders of La_2_O_3_ and Co_3_O_4_ using the floating-zone method. The LaCoO_3_/SrTiO_3_ thin film was fabricated using pulsed laser deposition in combination with *in situ* reflection high-energy electron diffraction (RHEED). It was grown under a 0.2 mbar O_2_ background pressure and at a deposition temperature of 750°C. The laser flux was adjusted to 1.9 J cm^−2^. The sample thickness was 55 nm which is determined by X-ray reflectivity (XRR) measurement. More sample details are given by Tomiyasu *et al.* (2017 ▸) and Wang *et al.* (2019 ▸).

The Co 2*p*3*d* RIXS measurements were performed at beamline BL05A1 at the Taiwan Light Source (Lai *et al.*, 2014 ▸). The scattering angle (α + β) was set at 140° for the LaCoO_3_ single crystal and at 90° for the LaCoO_3_/SrTiO_3_ film. Both samples were measured at the Co *L*_3_ edge (∼780 eV) at 20 K. The experimental resolution of RIXS was calibrated as ∼90 meV and the recorded incident energy broadening was ∼1000 meV (FWHM). The calculations of 2*p*3*d* RIXS spectra were performed using the program *Quanty* with intrinsic lifetime broadenings of ∼300 meV and 20 meV (FWHM) for the intermediate and final states, respectively (Haverkort *et al.*, 2012 ▸). In addition to the intrinsic lifetime broadening, we applied 1000 meV and 60 meV Gaussian broadening to simulate the experiment incident energy window and the energy loss resolution. However, the self-absorption effect depends on the intrinsic absorption factor (μ), which is independent of the instrumental broadening. Thus, the self-absorption-added coefficients in Fig. 3 ▸ include only intrinsic lifetime broadening. The experimental spectra were normalized to the fluorescence feature, where the fluorescence feature was subtracted from the normalized spectra (see also Appendix *B* [App appb]). The simulations were normalized to the charge transfer features.

## 4. Results and discussion   

**Figure 4:**
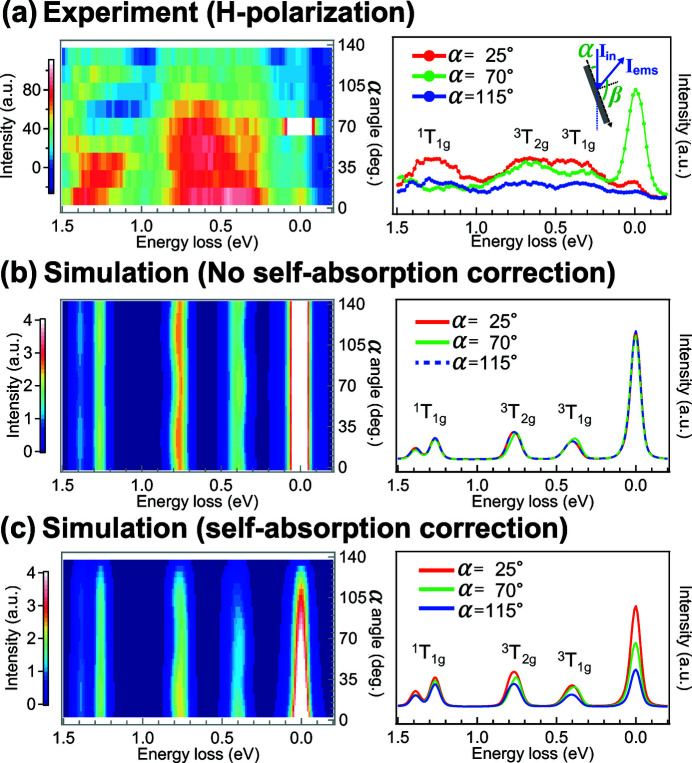
The 2*p*3*d* RIXS results as a function of rotation angle α (left panels) and slice cuts (right panels). (*a*) Experimental results of LaCoO_3_ single crystal. (*b*, *c*) Simulated spectra of ^1^ *A*_1*g*_(*O*_*h*_) ground state (*b*) without including and (*c*) including the saturation and the self-absorption effects. The scattering geometry of the calculation is identical to the angle used in the experiment on the LaCoO_3_ single crystal (α + β = 140°; horizontally-polarized incident photons).

Fig. 4(*a*) ▸ shows the angle-dependent RIXS of the LaCoO_3_ single crystal at the maximum of the Co *L*_3_ edge. The elastic peak shows a maximum intensity at the specular angle (β = α) due to strong reflection. In contrast, the intensity of the *dd* excitations decreases when α increases. In comparison, we show in Fig. 4(*b*) ▸ the α-dependent calculation of the ^1^A_1*g*_(*O*_*h*_) ground state, where the angular dependence can be observed. The ^3^T_1*g*_(O_*h*_) state slightly changes its intensity and shifts its position due to the competition of spin–orbit fine structures, which will be discussed later. However, the trends are not in good agreement with the experimental results because the saturation and the self-absorption effects should be considered in a concentrated sample. By including the saturation and the self-absorption effects, the multiplied results show good agreement for the trends of the angular dependence except that the elastic peak is overestimated [Fig. 4(*c*) ▸]. Although the spectra were distorted, the small energy shifts of the ^3^T_1*g*_ excited states are still preserved theoretically. Unfortunately, better statistics and energy resolution are required to observe this tiny shift in the experiment.

**Figure 5:**
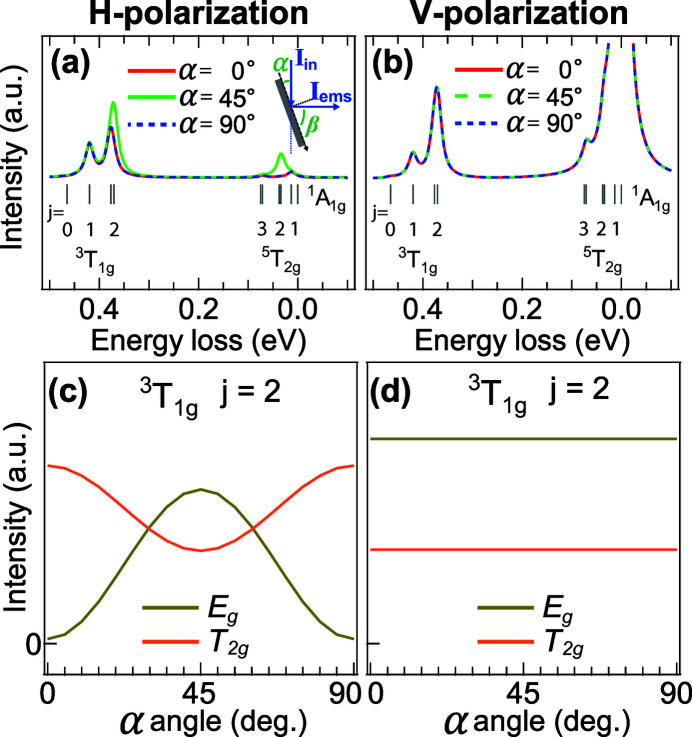
(*a*, *b*) 2*p*3*d* RIXS simulations of the ^1^ *A*_1*g*_(*O*_*h*_) ground state as a function of the sample rotation angle α with respect to (*a*) H-polarization and (*b*) V-polarization. Small broadening (20 meV) is used in the simulations. (*c*, *d*) 2*p*3*d* RIXS transition cross-section of the ^3^T_1*g*_ excited state as a function of the rotation angle α with respect to (*c*) H-polarization and (*d*) V-polarization. Presented here are only the states with a total moment equal to two (*j* = 2). The scattering angle is now replaced by 90°.

For an isotropic ^1^A_1*g*_ ground state, in general, no angular dependence in a dipole 2*p*3*d* transition (2*p* XAS) is observed (Merz *et al.*, 2010 ▸). However, the effective *dd* transition operator *W*_*Qq*_ determines the 2*p*3*d* RIXS cross-section, where the intermediate state plays an important role. This transition operator can be quadruple-like (rank 2 transition) because of the combination of two dipole transitions (rank 1 transition). On the one hand, the incident absorption channel of RIXS excites the ground state to intermediate states which preserve a mixture of different symmetries. On the other hand, due to the spin–orbit coupling, the final excited state splits into different branches with a small energy difference. In combination, the effective transition preserves spin and orbit characteristics and the polarization weighting factor *T*_*Qq*_(∊_E_, ∊_I_) provides the intensity of states as a function of geometry (van Veenendaal, 2006 ▸; Haverkort *et al.*, 2010 ▸). In order to confirm this idea, 20 meV resolved 2*p*3*d* RIXS simulations were performed. Figs. 5(*a*) and 5(*b*) ▸ show the 2*p*3*d* RIXS spectra of three geometries with the scattering angle (α + β) set to 90°. With a finer broadening, the energy splittings caused by spin–orbit coupling are better resolved [Figs. 5(*a*) and 5(*b*) ▸]. The ^3^T_1*g*_ states split into the *E*_*g*_, *T*_2*g*_, *T*_1*g*_ and *A*_1*g*_ states, where we have indicated the double-group labels in italic. In the case of vertically-polarized incident photons (V-polarization), the elements in the polarization tensor equally contribute in all geometries. Thus, no angular dependence is expected [*cf*. Fig. 5(*b*) ▸]. In contrast, the horizontally-polarized incident beam (H-polarization) induces the polarization tensor to change as a function of rotation angle. Thus, the angular dependence is pronounced and depends on the spin and orbit characteristics of the initial and the final state. Figs. 5(*c*) and 5(*d*) ▸ present the angle-dependent behavior of different polarizations by plots of the RIXS cross-section probed at the maximum of the *L*_3_ edge as a function of rotation angle. Here the *E*_*g*_ and *T*_2*g*_ groups of the ^3^T_1*g*_ state are selected for comparison. We note that the broadening of the spectra in Fig. 4 ▸ has been increased due to the limiting experimental resolution. Hence the angular dependence reflects the intensity competition and energy shift.

**Figure 6:**
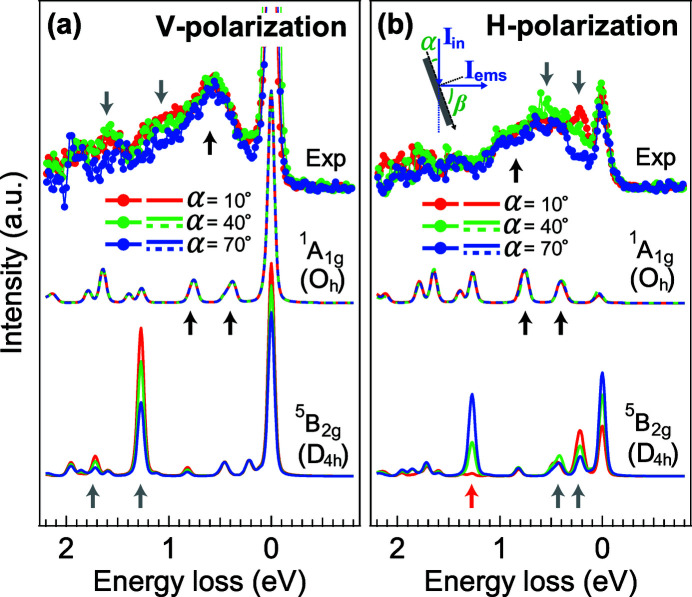
The 2*p*3*d* RIXS results of the 55 nm LaCoO_3_/SrTiO_3_ film compared with the ^1^A_1*g*_(O_*h*_) and ^5^B_2*g*_(D_4*h*_) ground states. Both the (*a*) V- and (*b*) H-polarized are calculated and measured at a geometry for a scattering angle of 90°.

One way to avoid the self-absorption effect is by reducing the sample thickness, so that the emission intensity is an energy-independent function of the rotation angle [*cf*. equation (10)]. Once we normalize the spectra to the fluorescence feature, the intensity of the *dd* excitations can be represented properly. We examine the angle-dependent 2*p*3*d* RIXS of the 55 nm LaCoO_3_ film grown on the SrTiO_3_ substrate. This thickness is on the limit of the attenuation length of the Co *L*_3_ edge (25–125 nm) but can still be considered to be free from self-absorption effects (see further arguments in Appendix *A* [App appa]). The *dd* excitations are now less influenced by the self-absorption effect and are mainly determined by the polarization selectivity – see Fig. 6 ▸. Most of the angle-dependent features agree with the calculation of the ^5^B_2*g*_(D_4*h*_) ground state (gray arrows). As has been discussed, the ground state of the Co^3+^ ions in the LaCoO_3_/SrTiO_3_ film shows a mixture of the low-spin ^1^A_1*g*_(O_*h*_) state and high-spin ^5^B_2*g*_(D_4*h*_) state (Wang *et al.*, 2019 ▸). Compared with the ^5^B_2*g*_(D_4*h*_) state, the angular dependence of RIXS is negligible for the ^1^A_1*g*_(O_*h*_) state (Fig. 6 ▸). The angle-independent features of the ^1^A_1*g*_(O_*h*_) state are indicated by black arrows, but are almost invisible compared with the ^5^B_2*g*_(D_4*h*_) ground state. Some discrepancies remain, for example the 1.3 eV feature indicated with the red arrow in the ^5^B_2*g*_(D_4*h*_) calculation is not evident in the experimental data. This could be because: (i) the mixture of the spin states suppresses the intensity of the 1.3 eV feature (Wang *et al.*, 2019 ▸); (ii) we assumed that the CoO_6_ clusters are well aligned to the pseudo-cubic orientation (002) – experimentally, not all the CoO_6_ clusters should be aligned exactly along the pseudo-cubic orientation; (iii) the discussions above do not take the trigonal symmetry reduction into account. The CoO_6_ clusters are naturally aligned in a trigonal field for a relaxed LaCoO_3_ crystal (the space group of the unit cell is 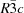). By including the trigonal distortion, the polarization selectivity might be different from the tetragonal distortion.

## 5. Conclusions   

We have presented the angular dependence of 2*p*3*d* RIXS analyzed with the scattering cross-section including saturation and self-absorption effects. The angle-dependent spectra are a better probe of the symmetry type than using only the difference between two orthogonal polarizations, due to the angular relation between the ground/excited states symmetry and the polarization tensor. A remarkable consequence is that even the isotropic low-spin ^1^A_1*g*_(O_*h*_) ground state shows angle-dependent 2*p*3*d* RIXS spectra, despite the absence of angular dependence in the 2*p* XAS spectra (Merz *et al.*, 2010 ▸; Wang *et al.*, 2019 ▸). Unfortunately, the high Co concentration in the LaCoO_3_ single crystal distorts the RIXS spectra due to the saturation and self-absorption effects which limits the distinguishability. By applying a model to consider these effects, the trends are explained as a function of both the energy and the rotation angle. In contrast, for the LaCoO_3_/SrTiO_3_ film, the self-absorption effect is small. Thus the LaCoO_3_/SrTiO_3_ film shows better agreement with the angular trend although some features are not reproduced well. The possible reason for this mismatch could be a mixture of ground states, non-perfect empirical parameters and/or reduced symmetry.

## Figures and Tables

**Figure 7:**
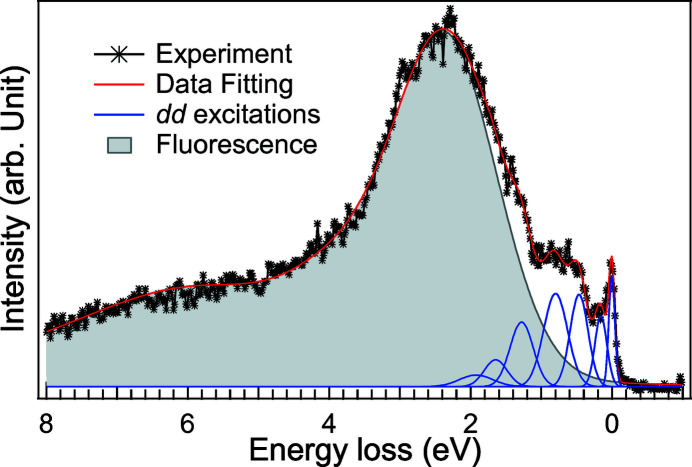
Fitting treatment of the RIXS spectra for LaCoO_3_/SrTiO_3_ film. Blue peaks are the Gaussian fitting profiles of *dd* excitation features. The gray area is the fitting profile for the fluorescence feature.

## Appendix A Estimation of the attenuation length of the elements at the Co *L*_3_ edge (∼780 eV)

In the main text, we use the relative relation of the absorption factor (μ) to describe the trends of the saturation and self-absorption effects. Here we provide the experimental quantities of different elements in the LaCoO_3_ to estimate the following questions: (*a*) What is the maximum attenuation length of the Co *L*_3_ edge? (*b*) What is the critical film thickness in order to claim the absence of the self-absorption effect? (*c*) What is the reasonable weighting for μ_B_?

According to the tabulated data (Henke *et al.*, 1993 ▸), the attenuation length at 780 eV for the La, O and Co elements in the LaCoO_3_ (ρ = 7.25 g cm^−3^) are ∼950, ∼810 and ∼380 nm, respectively. The values show that the attenuation length of Co is much longer than our film thickness (55 nm), which leads to the consequence that our film can be self-absorption free.

However, the attenuation length values at the absorption edge are likely overestimated using Henke’s table. For cobalt metal (ρ = 8.9 g cm^−3^), the estimated attenuation length is ∼75 nm but the experimental results indicate that the attenuation length was ∼25 nm at the peak maximum (Chen *et al.*, 1995 ▸). By considering that the mass weight of Co is about 25% in the LaCoO_3_ (ρ_Co_ = 1.8 g cm^−3^), the attenuation length of the Co *L*_3_ edge peak maximum is ∼125 nm (five times larger). This value is still larger than our film thickness of ∼55 nm.

On the other hand, the self-absorption effect on the RIXS profile will be dependent on the intrinsic lifetime broadening. If we set a lifetime broadening of 300 meV, the selected energy at a few hundred meV away from maximum of the *L*_3_ resonance peak has an attenuation length a few times longer. This implies that we can treat the 55 nm LaCoO_3_ film as being free from self-absorption effect at the orbital excitations (>200 meV). However, we remark that minor self-absorption effects could be visible for the elastic and low-loss features (<150 meV).

Here we estimate the background absorption. The weighting of μ is proportional to the inverse of the attenuation length. It gives values of 2–10%, 3–12% and 95–78% with respect to elements La, O and Co (attenuation lengths are 950, 810 and 25–125 nm), respectively. The background absorption factor (μ_B_) is the off-resonant contribution from the other elements (except Co) and is estimated to be about 5–22%. We set μ_B_ as ∼5% of μ_max_ in our model.

## Appendix B RIXS spectra normalization

Fig. 7 ▸ presents our treatment on the RIXS spectrum of the LaCoO_3_/SrTiO_3_ film. The blue peaks are the *dd* excitations which were fitted by Gaussian functions, grouped with the elastic peak at 0 eV, low-energy peaks at ∼0.1–0.2 eV and the other excitations at about 0.4, 0.8, 1.3, 1.5 and 2.0 eV. The broadening is increased from 0.1 to 0.4 eV (FWHM) with the increase of the energy loss.

In order to compare the calculation with the experimental results, we normalized the spectra to the summed area of the charge transfer/fluorescence features. These features (>2 eV) are expected to be self-absorption free according to our discussion. We use five Gaussian functions to simulate the experimental fluorescence profile (gray area in Fig. 7 ▸). This fluorescence profile was used for the normalization procedure. After the spectra were normalized, the fluorescence profile was subtracted from the normalized spectra.
